# Progress in quantum information

**DOI:** 10.1093/nsr/nwaf289

**Published:** 2025-07-16

**Authors:** Gui-Lu Long

**Affiliations:** Department of Physics, Tsinghua University and State Key Laboratory of Low-dimensional Quantum Physics, China; Beijing Academy of Quantum Information Sciences, China; Frontiers Center of Quantum Information, Ministry of Education, China

This year marks the 100th anniversary of the establishment of quantum mechanics. Evolving from a purely theoretical framework, it has grown into a thriving scientific discipline. Among its most significant contemporary developments is quantum information science, which emerged in the 1980s. Comprising three primary branches—quantum computing, quantum communication, and quantum sensing and measurement—this field faces substantial challenges, several of which are addressed in this special topic of the *National Science Review*.

Quantum communication utilizes quantum states as carriers of random numbers or information. It enables detection of eavesdropping in communication through the fundamental principles of quantum mechanics. As a form of quantum state measurement, eavesdropping induces state changes, thereby causing a significant increase in quantum bit error rates. Wang and Long provide a concise overview of recent advancements in quantum secure direct communication (QSDC), a technology that directly transmits information via quantum states [1]. Beyond improvements in distance and transmission rate, QSDC is being integrated with traditional networks to form hybrid quantum-classical networks, alongside the development of authentication protocols. Zhang *et al*. report their recent progress in polarization-encoded quantum key distribution using a room-temperature telecom single-photon emitter, highlighting the potential of GaN defects in supporting polarization-encoded quantum communication [2].

Quantum sensing and measurement leverage the properties of quantum states to achieve more precise and accurate measurements of various physical quantities, forming a substantial portion of this special issue. Lei *et al*. focus on the sensitivities of quantum magnetic sensing [3]. Wu *et al*. present experimental progress in investigating non-Hermitian physics within the quantum regime, with particular emphasis on exceptional points [4]. Shen and Zhang describe advancements in entanglement-enhanced quantum metrology using neutral atom arrays [5]. Tong *et al*. report cutting-edge research on the operation of thorium nuclear optical clocks [6].

The review by Chen *et al*. offers a comprehensive analysis of time–frequency transfer over optical fibers [7]. Optical time–frequency transfer establishes the metrological linkage in large-scale clock networks, which facilitates various applications. The review provides an overview of the advances in optical two-way time–frequency transfer, including system configuration, key modules, main challenges, and mainstream transfer methods. They give an outlook on further applications toward global-scale high-precision clock networks.

The integration of quantum science and artificial intelligence has yielded productive outcomes. Ma *et al*. review the application of machine learning in the estimation and control of quantum systems [8]. This paper examines key topics at the intersection of machine learning and quantum estimation and control, including neural network-based approaches for quantum state estimation, gradient-based methods for quantum optimal control, evolutionary computation for learning control of quantum systems, machine learning techniques for robust quantum control, and reinforcement learning for adaptive quantum control.

Superconducting quantum computing stands as the leading technology in the field. Jiang *et al*. review advancements in this area, detailing fabrication methodologies for superconducting quantum chips and implementations of quantum gate operations [9]. The review synthesizes experimental progress in superconducting quantum computing over recent years, encompassing the preparation of multi-qubit entangled states, random circuit sampling experiments, demonstrations of quantum error correction based on surface codes, error mitigation techniques, and quantum simulations. It also discusses experimental advancements related to bosonic-encoded qubits, fluxoniums, and qudits. Finally, current challenges in scaling are analyzed, and potential solutions to these limitations are explored.

In summary, quantum information processing is advancing rapidly across a broad range of fields. This special topic covers only a subset of these advancements, with many more unfolding as of this writing. We aim to encompass these additional developments in future issues.
